# Protective effects of cannabidiol against acute stress: behavioral responses and corticosterone concentrations in female Wistar rats

**DOI:** 10.3389/fphar.2026.1931847

**Published:** 2026-09-30

**Authors:** Matjaž Uršič, Mina Cansu Karaer, Alenka Gregorič, Tilen Vake, Tomaž Snoj

**Affiliations:** 1 Veterinary Faculty, Institute of Preclinical Sciences, University of Ljubljana, Ljubljana, Slovenia; 2 A-vet Sežana, Sežana, Slovenia

**Keywords:** anxiolytic-like effect, cannabidiol, corticosterone, female Wistar rats, open field test

## Abstract

**Background:**

Cannabidiol (CBD) has been widely investigated for its potential anxiolytic and stress-modulating effects. This study evaluated whether CBD reduces the stress response induced by acute restraint stress in rats.

**Methods:**

Thirty-six female Wistar rats were randomly assigned to six groups. Four experimental groups received CBD at different doses (2, 10, 20, and 100 mg/kg), while two control groups received the placebo (CBD vehicle medium-chain triglycerides). Sixty minutes after administration, the animals treated with CBD and one placebo-treated group (stressed control group) were subjected to 60 min of restraint stress. The other placebo-treated group (nonstressed control group) was not subjected to restraint stress. Subsequently, all animals were tested in the open field (OF) for 30 min. Immediately after the OF test, blood samples were collected for corticosterone analysis. Plasma corticosterone concentrations were determined using a commercial ELISA kit.

**Results:**

In the OF, distance traveled differed significantly between groups, although no significant differences were observed among the different CBD doses. Compared with stressed control rats, CBD-treated rats spent more time in the center of the OF arena, suggesting reduced anxiety-like behavior. Plasma corticosterone concentrations did not differ significantly between groups. At 90 min after the onset of stress (30 min after the end of restraint stress), corticosterone concentrations did not differ between CBD-treated and untreated rats.

**Conclusion:**

CBD showed no effect on locomotor activity but increased center time, suggesting anxiolytic-like effects. Further studies are needed to clarify the underlying mechanisms.

## Graphical Abstract

**Figure:**
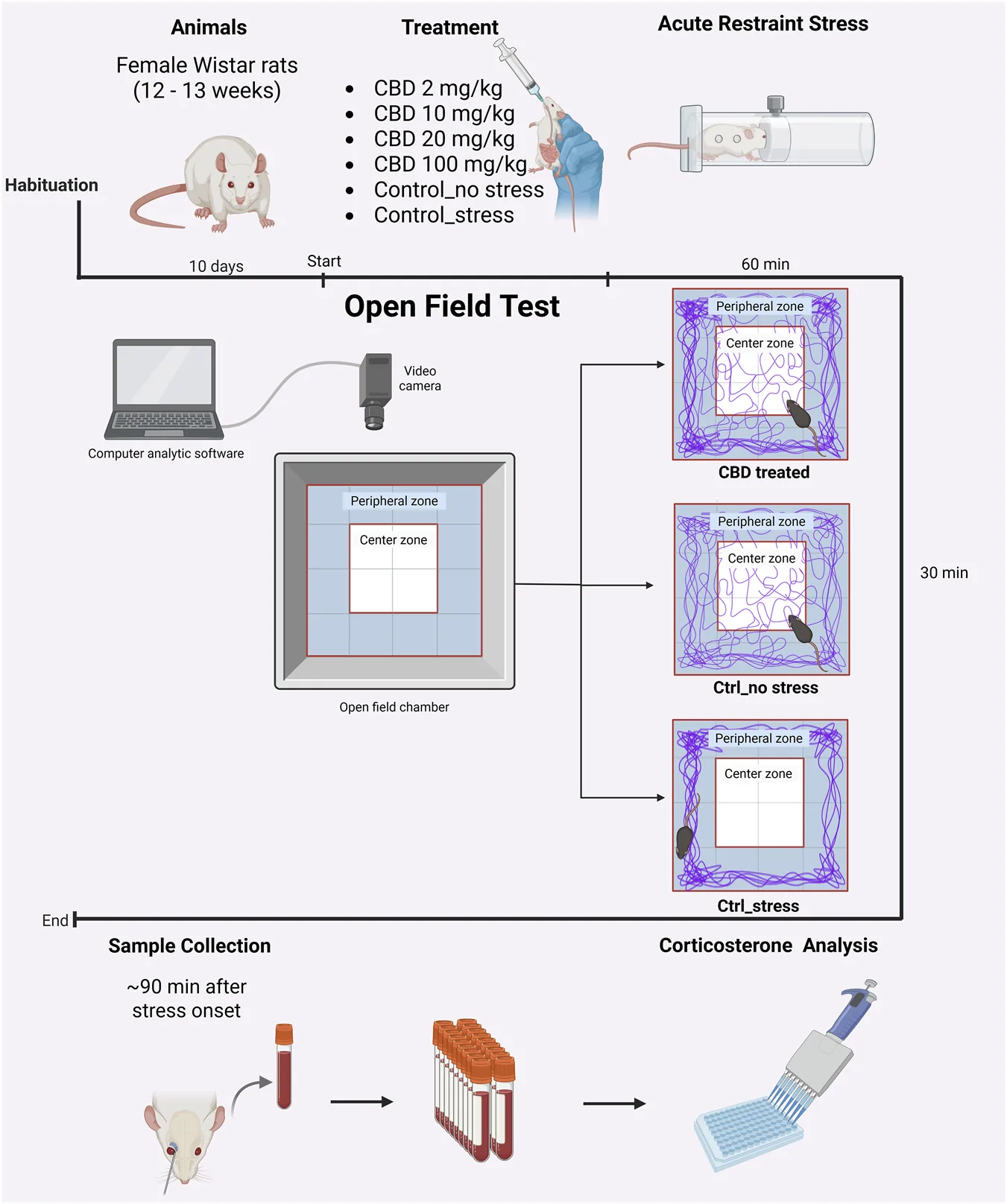

## 1 Introduction

Cannabidiol (CBD) has been increasingly investigated for its anxiolytic and stress-modulating properties at preclinical and clinical levels. Evidence suggests that CBD influences anxiety-related behaviors through multiple neurobiological pathways, including modulation of the endocannabinoid system (ECS) and serotonergic signaling, particularly via 5-HT1A receptors (Shu et al., 2024; Campos et al., 2012; Zuardi et al., 2009; Zuardi et al., 2017; Brancato et al., 2023).

The ECS plays a key role in the modulation of stress and emotional responses; it comprises endocannabinoids such as anandamide (AEA) and 2-arachidonoylglycerol (2-AG), the cannabinoid receptors CB1 and CB2, and the enzymes responsible for their synthesis and degradation. Endocannabinoids are produced on demand and act as retrograde neurotransmitters, modulating synaptic transmission (Freund et al., 2003; Lu and Mackie, 2016). CB1 receptors are abundantly expressed in brain regions critically involved in stress processing and emotional regulation, including the amygdala, hippocampus, prefrontal cortex, and other limbic structures (Freund et al., 2003). Through these pathways, the ECS is implicated in the regulation of anxiety-related behavior, stress-coping strategies, and hypothalamic‒pituitary‒adrenal (HPA) axis activity (Gupta et al., 2024; Șerban et al., 2025), providing a functional framework for cannabinoid action.

Within this framework, CBD has a complex pharmacological profile characterized by low affinity for CB1 and CB2 receptors and an indirect mechanism of action; its anxiolytic and stress-modulating effects are primarily attributed to the facilitation of serotonergic neurotransmission via agonistic action at 5-HT1A receptors, through the inhibition of enzymes that metabolize anandamide, such as fatty acid amide hydrolase (FAAH), and the modulation of the limbic circuitry involved in emotional processing (Campos et al., 2012). In addition to binding to cannabinoid receptors, endocannabinoids and phytocannabinoids interact with noncannabinoid targets such as serotonin 5-HT1A receptors, transient receptor potential vanilloid 1 (TRPV1) channels, peroxisome proliferator-activated receptor gamma (PPARγ), and regulate Ca2+ mobilization further expanding their role in stress modulation, and synaptic plasticity (Eldridge and Landfield, 1990; Costa et al., 2004; Wang et al., 2012; Lu and Mackie, 2016; Campos et al., 2012; Ryan et al., 2009; Brancato et al., 2023).

Beside these mechanistic effects, the influence of CBD on the HPA axis was also reported. While human studies report anxiolytic effects of CBD, with endocrine outcomes remaining less consistently characterized. In rodents, CBD has been shown to effectively modulate behavioral and autonomic responses (Resstel et al., 2009; Lisboa et al., 2024), and to reduce corticosterone concentrations following the Δ9-tetrahydrocannabinol exposure (DeVuono et al., 2022) or independently of previous alcohol consumption (Brancato et al., 2023). However CBD administration itself has been reported to increases blood corticosterone concentration (Zuardi et al., 1984). Consistent with this observation, experimental studies in rodents have demonstrated that CBD reduces anxiety-related behaviors, such as stress-induced hyperthermia and related avoidance or immobility behavior responses, while affecting behavioral and neurochemical correlates associated with these phenotypes (Shu et al., 2024). For these experimental studies, the behavioral effects of CBD have been extensively evaluated using validated rodent assays, including the open field (OF) test, elevated plus maze, light/dark box, forced swim and tail suspension tests, and stress-induced hyperthermia models.

These approaches allow the assessment of anxiety-like behavior, exploratory activity, and stress-related behavioral and endocrine responses, with several studies demonstrating that CBD attenuates anxiety-like behaviors across these models (Saad et al., 2023; Shu et al., 2024; Jantsch et al., 2025; Jîtcă et al., 2025; Lucindo et al., 2025; Zlatanova-Tenisheva et al., 2025; Jenkins et al., 2026). In rodent stress-evaluating models, CBD has been shown to reduce stress-related behavioral alterations. Under chronic stress conditions such as social isolation, CBD decreases anxiety- and depression-like behaviors, as reflected by reduced immobility and increased exploratory activity (Saad et al., 2023; Lucindo et al., 2025). In predator-based models, however, the effects are less consistent (Jîtcă et al., 2025). In acute and metabolic stress models, CBD has also been shown to attenuate stress-induced behavioral responses, including reduced anxiety-like behavior and improved stress-related behavioral outcomes in standard tests such as the OF and elevated plus maze (Zlatanova-Tenisheva et al., 2025; Jantsch et al., 2025). CBD additionally modulates behavior in nonstress models, such as the Black and Ten Brachyury (BTBR) mouse model of autism, in which it acts as a dose-dependent behavioral modulator, rescuing social deficits at lower doses (20 mg/kg) and reducing repetitive grooming and hyperlocomotion at higher dose (50 mg/kg) during long-term exposure (Shrader et al., 2024).

Based on the existing literature, our study aims to address gaps in the knowledge of CBD effects in laboratory animals. Most previous studies have used males (Campos et al., 2012; Shu et al., 2024; Lucindo et al., 2025; Zlatanova-Tenisheva et al., 2025; Jantsch et al., 2025; Ruanpang et al., 2025) and the effects of CBD on acute stress in females remain unexplored. In addition, studies assessing the dose-depending effects of CBD have shown an inverted U-shaped response (Resstel et al., 2009; Fabris et al., 2022; Uršič et al., 2022; Shrader et al., 2024), which was also of interest in the present study, particularly with regard to variability in behavioral response. Also, the simultaneous assessment of behavioral responses and plasma corticosterone concentrations in a dose-response design may provide a more comprehensive characterization of CBD’s effects on the stress response.

On the basis of these considerations, the aim of the present study was to investigate whether CBD premedication diminishes the intensity of the stress response induced by acute movement restriction in female rats. Stress-related behavior was assessed using the OF test, and plasma corticosterone concentrations were determined as an indicator of HPA axis activation.

## 2 Materials and methods

### 2.1 Animals and animal procedure

The animal procedures were approved by the Administration of the Republic of Slovenia for Food Safety, Veterinary Sector and Plant Protection (license No. U34401-15/2019/8).

Thirty-six female Wistar rats, provided by Inotiv Italy (Udine, Italy), were used in this study. Upon arrival at our laboratory, the rats were 11–12 weeks of age. The rats were randomly divided into six groups with six animals in each group and housed under standard laboratory conditions: the temperature was maintained at 22 °C ± 2 °C, with relative humidity ranging from 30% to 70%, and artificial lighting was provided on a 12-h light/12-h dark cycle (light phase was from 6 a.m. to 6 p. m). Water and a conventional laboratory rodent diet produced by Altromin (Lage, Germany) were provided *ad libitum*. The animals were housed in groups of six in polypropylene cages measuring 66 cm × 40 cm × 20 cm. The animals were allowed to acclimate to the laboratory conditions for 10 days before the start of the experimental procedure. At the time of the procedure, the animals were 12–13 weeks of age.

To ensure comparable absorption of CBD from the gastrointestinal tract, food was withheld for 3 h prior to starting the animal procedure, while drinking water remained available *ad libitum*.

Cannabidiol (98.5% purity; Vakos XT, Prague, Czech Republic) dissolved in medium-chain triglyceride (MCT) oil at a concentration of 100 mg/mL was purchased from the Pharmacy of the University Medical Center Ljubljana (Ljubljana, Slovenia). The CBD was manufactured according to Good Manufacturing Practice (GMP), and the Certificate of Analysis, including cannabinoid composition and purity data, is provided in Supplementary Material S1. Before administration, CBD was further dissolved in MCT oil to reach the concentrations shown in Table 1. CBD was administered via orogastric gavage into the stomach at a dose of 0.5 mL per 100 g of body weight. The CBD doses are listed in Table 1. Doses were determined on the basis of previously published studies with the highest dose (100 mg/kg) included to explore the possibility of an inverted U-shaped dose-response relationship (Zuardi et al., 2017; Fabris et al., 2022; Uršič et al., 2022; Shrader et al., 2024; Ruanpang et al., 2025). The group receiving 0 mg/kg received only MCT oil, which served as the solvent and control. After administration, the animals were returned to their home cages for 60 min. Afterward, stress was subsequently induced by restricting animal movement for 60 min using a restraint device (homemade by local manufacturer, Supplementary Material S2). All animal procedures were performed at the end of the light phase of the light/dark cycle, between 2:00 p. m. and 6:00 p. m. The arena was illuminated with artificial light at an intensity of 200–210 lux.

**TABLE 1:** Administered doses of CBD and movement restriction for individual groups of rats.

Group name	2 mg/kg (n = 6)	10 mg/kg (n = 6)	20 mg/kg (n = 6)	100 mg/kg (n = 6)	Control nonstressed (n = 6)	Control stressed (n = 6)
CBD dose (mg/kg)	2	10	20	100	0	0
Administered CBD concentrations (mg/mL)	0.4	2.0	4.0	20.0	0.0	0.0
Induced stress (movement restriction)	Yes	Yes	Yes	Yes	No	Yes

Immediately after restraint, each animal was transferred directly to the OF arena (100 cm × 100 cm), which was surrounded by a 40 cm high fence. The animal was placed in the middle of the OF arena. Spontaneous behavior was recorded for 30 min. To minimize the influence of olfactory cues between consecutive trials, the surface of the OF arena was thoroughly cleaned with 70% ethanol after each test.

Behavioral analysis focused on parameters commonly associated with stress and anxiety-related responses in rodents. This analysis included the total distance traveled and time spent in the central zone of the OF arena. Automated analyses of locomotor activity parameters were performed using ToxTrack software (Umeå University, Umeå, Sweden) by reviewing video recordings.

Following completion of the behavioral assessment, general anesthesia was induced using an inhalation anesthesia system (Eickemeyer, Sunbury-on-Thames, United Kingdom) with 4% isoflurane (Isoflurin, Vetpharma Animal Health, Barcelona, Spain). During general anesthesia, blood samples (500 µL) were collected via puncture of the retro-orbital venous sinus using heparinized microhematocrit capillary tubes (Euroglass, Ljubljana, Slovenia). The blood was transferred into tubes containing the anticoagulant K3EDTA. Blood samples were centrifuged at 2,500 × g, and the plasma was removed and stored at −20 °C until analysis of the corticosterone concentration. After recovering from general anesthesia, the animals were housed in home cages and subsequently used for other procedures.

Plasma corticosterone concentrations were measured using a commercially available enzyme-linked immunosorbent assay (ELISA) kit (Corticosterone Rat/Mouse ELISA Kit, Demeditec, Kiel, Germany) specifically validated for rats and mice, following the manufacturer’s instructions.

### 2.2 Statistical analysis

A prior power analysis (80% power, α = 0.05) was performed based on data from a pilot study, in which the control group had a mean time spent in the center of the open field of 22.4 with a standard deviation of 2.6. Assuming a 20% difference between a control and treatment groups, the analysis indicated that a minimum of five animals per group was required. To account for potential attrition, six animals were included in each experimental group. Sample size calculations were performed using ClinCalc Sample Size Calculator (https://clincalc.com/Stats/SampleSize.aspx). Data analysis was conducted using the R statistical programming language (https://www.r-project.org) in the RStudio environment (version 2026.01.1, Build 403). Behavioral and biochemical outcomes were analyzed using linear models (LMs) with Group as the predictor. Model assumptions were assessed by visual inspection of residual plots for normality and homogeneity of variance. When these assumptions were violated and the data were positively skewed, generalized linear models (GLMs) with a gamma distribution and log link were applied. Statistical significance was evaluated using analysis of variance (LM) or analysis of deviance (GLM) with F tests. Estimated marginal means were obtained using the *emmeans* package and used for Tukey-adjusted pairwise comparisons. Statistical significance was set at α = 0.05. Effect sizes are reported as eta-squared (η^2^) for linear models and Nagelkerke’s pseudo-R^2^ for generalized linear models. The results are plotted as estimated marginal means with 95% confidence intervals, along with individual data points and results of pairwise between-group comparisons.

## 3 Results

Behavioral tests and corticosterone concentrations were evaluated using different regression models. The time spent in the center of the open field test arena showed positively skewed distributions and were therefore analyzed using GLMs with a gamma distribution and log link; LMs were used for all other outcomes. Group had moderate to large effects on most outcome measures, as summarized in Table 2. Significant effects of Group were observed for distance traveled and center time; corticosterone levels were not significantly affected. Detailed summaries of all models and the *post hoc* tests are provided in the Supplementary Material S3.

**TABLE 2:** Summary of model results for behavioral and biochemical outcomes.

Outcome	Model	Statistic	*p* value	Effect size
Center time	Gamma GLM	F(5, 30) = 5.2	0.002	Pseudo *R* ^2^ = 0.48
Distance traveled	LM	F(5, 30) = 7.9	<0.001	η^2^ = 0.53
Corticosterone	LM	F(5, 30) = 2.0	0.104	η^2^ = 0.25

### 3.1 Open field test

#### 3.1.1 Distance traveled

Distance travelled differed significantly between groups (*F*(5, 30) = 7.9, *p* < 0.001, η^2^ = 0.53). Ascending doses of CBD did not clearly affect distance traveled in the open field test in a dose-dependent manner. Distance traveled was generally comparable across treatment groups, with no significant differences observed between CBD doses. In contrast, compared with rats in several other groups, rats in the control nonstressed group traveled significantly shorter distances, indicating reduced activity (Figure 1).

**FIGURE 1:**
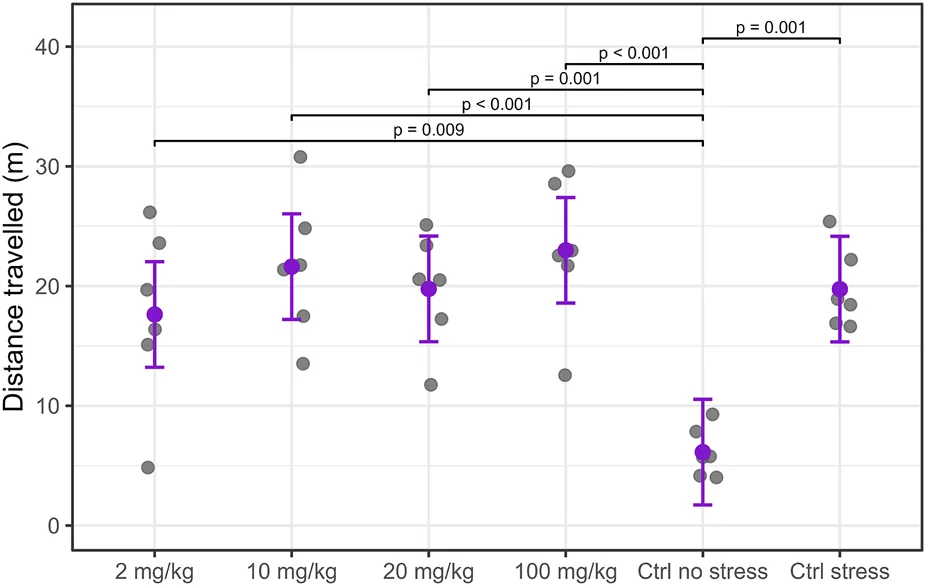
Group differences in distance traveled inside the open field test arena. Marginal estimated means with corresponding 95% confidence intervals are shown in purple, and individual observations are shown as gray circles. CBD doses administered to stressed rats are expressed in mg/kg. ‘Ctrl no stress’ represents animals in the control group that received no CBD and were not subjected to restraint stress, whereas ‘Ctrl stress’ represents animals in additional control group that received no CBD but were subjected to restraint stress.

#### 3.1.2 Center time

Time spent in the center of the open field arena differed significantly between groups (Gamma GLM: *F*(5, 30) = 5.2, *p* = 0.002, Nagelkerke *R* ^2^ = 0.48). Group effects on time spent in the center of the OF test arena did not follow a clear monotonic dose‒response pattern, as shown in Figure 2. The time spent in the center increased across the lower CBD doses, whereas relative to intermediate doses, the highest dose resulted in reduced values. Compared with rats in several other groups, rats in the Ctrl stress group spent significantly less time in the center. The variability within this group appeared lower than that in the other groups.

**FIGURE 2:**
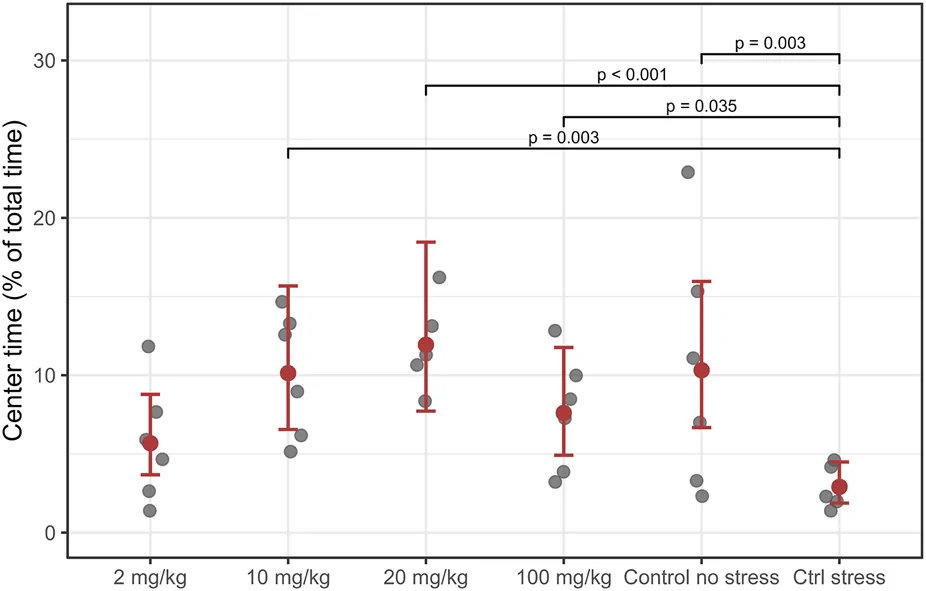
Group differences in time spent in the center of the open field test arena. Marginal estimated means with corresponding 95% confidence intervals are shown in red, and individual observations are shown as gray circles. CBD doses administered to stressed rats are expressed in mg/kg. ‘Ctrl no stress’ represents animals in the control group that received no CBD and were not subjected to restraint stress, whereas ‘Ctrl stress’ represents animals in additional control group that received no CBD but were subjected to restraint stress.

### 3.2 Corticosterone concentration

Serum corticosterone concentrations did not differ significantly between groups (*F*(5, 30) = 2.0, *p* = 0.104, η^2^ = 0.25). Compared with the other groups, both control groups presented slightly lower values, although these differences were not statistically significant (Figure 3).

**FIGURE 3:**
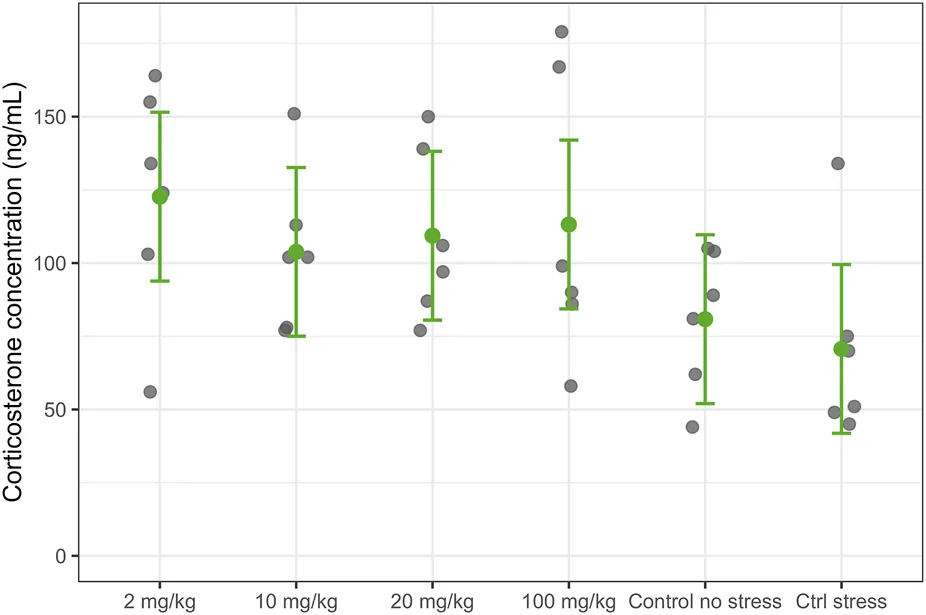
Group differences in plasma corticosterone concentrations. Marginal estimated means with corresponding 95% confidence intervals are shown in green, and individual observations are shown as gray circles CBD doses administered to stressed rats are expressed in mg/kg. ‘Ctrl no stress’ represents animals in the control group that received no CBD and were not subjected to restraint stress, whereas ‘Ctrl stress’ represents animals in additional control group that received no CBD but were subjected to restraint stress.

## 4 Discussion

In this study, we investigated the effects of CBD on behavioral and corticosterone responses to induced acute stress in Wistar rats. Female Wistar rats were selected because the effects of CBD as an anxiolytic agent have been less extensively investigated in rat females than in males, while Wistar rats are a well-established laboratory model for assessing anxiety-related behavior and the anxiolytic effects of pharmacological agents using the OF test (Bert et al., 2001; Almeida et al., 2010; Klenerova et al., 2011). The results of our study indicate an unexpected pattern of behavior in the OF test. Compared with nonstressed non-CBD-treated control rats, rats receiving CBD and being exposed to stress displayed increased movement activity, resulting in a longer total distance traveled. In contrast, non-CBD-treated stressed animals showed locomotor activity comparable to that of CBD-treated stressed animals (Figure 1). Contrary to initial expectations, the highest level of exploration was not observed in the nonstressed group, which would typically be anticipated under baseline conditions.

In the OF paradigm, total distance traveled is typically interpreted as a direct indicator of general locomotor activity and, indirectly, of anxiety-related behavior. Under normal circumstances, acute restrain stress is expected to reduce exploratory behavior and promote reduced locomotor activity (Trneckova et al., 2004; Klenerova et al., 2011), and anxiolytic interventions such as CBD pretreatment are hypothesized to prevent stress-induced suppression of activity (Carli et al., 1989).

The results of our study are somewhat contradictory and do not match the generally accepted paradigm of the interpretation of anxiety-like behavior in the OF test. It was expected that nontreated nonstressed animals would express more exploratory behavior than those that were stressed. Surprisingly, the opposite results were obtained in our study. Moreover, all stressed animals, regardless of whether they were previously treated with CBD or not, showed comparable total distance traveled, and the distance travelled by all of them was significantly higher than in the nonstressed nontreated animals (Figure 1). On the one hand, the results show that the anxiolytic effect of CBD was not observed (since there was no difference between stressed treated and nontreated animals). On the other hand, the results of the stress-related behavior, expressed as total distance traveled, revealed that nonstressed animals exhibited lower locomotor and exploratory activity than both stressed treated and stressed untreated animals did. This observation is contrary to findings commonly reported in previous studies (Carli et al., 1989; Prut and Belzung, 2003; Trneckova et al., 2004). The inclusion of nonstressed CBD treated group would have provided additional insight into the observed findings. Specifically, it would have allowed us to distinguish whether the reduction in exploratory behavior was attributable to induced stress, CBD treatment, or their interaction. However, at the time the study protocol was designed a reduction in exploratory behavior in nonstressed untrated animal was not anticipated. Therefore, nonstressed CBD treated group was not included in the experimental design. In retrospect, the absence od this group should be concidered as a limitation of the present study. Moreover, previous research has suggested that the anxiolytic effects of CBD may differ between males and females and may be influenced by the stage of the estrous cycle in females, with no significant anxiolytic effects observed during proestrus (Fabris et al., 2022). Such sex- and cycle-dependent variability could potentially contribute to the heterogeneous behavioral responses observed in our study and highlights the need for further investigation. During our study, the phase of the estrus cycle was not controled. Although animals were randomly assigned to the OF assessment on each testing day, this somehow distribute animals in different phase of estrus cycle over the testing period. However the phase of the estrous cycle may have contributed to variability in behavioral responses. Fluctuations in ovarian hormones, particularly estradiol and progesterone, can influence locomotor activity, exploratory behavior, and anxiety-like behavior and may therefore affect parameters measured in the open-field test, including distance traveled and time spent in the central area (Miller et al., 2021). Moreover, the behavioral effects of CBD may themselves be modulated by the hormonal status of females, potentially contributing to variability in the observed response (Fabris et al., 2022). In addition, fluctuations in ovarian hormones may influence corticosterone responses, as they modulate HPA-axis activity and stress-induced corticosterone secretion in female rats (Atkinson and Waddell, 1997). Since all of these mechanisms are not fully understood yet, future studies could control for the estrous cycle or use ovariectomized animals to reduce the influence of fluctuating ovarian hormone levels, although ovariectomy would represent a different hormonal state and should therefore be considered when interpreting the results.

In addition, Ruanpang et al. (2025) conducted a study in which CBD-treated rats were subjected to 3-hours of restraint stress daily for28 consecutive days. The authors included the OF test and reported that compared with the control, CBD did not significantly increase locomotor activity in the OF test. Therefore, our findings and the findings of Ruanpang et al. (2025) suggest a more complex interaction between stress exposure and CBD treatment, that cannot be explained completely through the interpretation of OF test results. One possible explanation is that stress alone may have induced a ceiling or compensatory effect on locomotor activity, leading to behavioral adaptation rather than suppression. However, further studies are needed to confirm this hypothesis. Alternatively, the lack of a difference among the stressed groups, regardless of CBD administration, may indicate that the stress protocol used was insufficient to produce a measurable reduction in exploratory activity, thereby masking potential CBD-related effects.

The increased locomotion observed in the CBD-treated stressed group compared with that in the nonstressed control group may also reflect a context-dependent modulation of arousal rather than an anxiolytic effect. CBD is known to interact with multiple neurobiological systems, including serotonergic and vanilloid pathways (Costa et al., 2004; Wang et al., 2012; Lu and Mackie, 2016; Campos et al., 2012) which may differentially influence arousal, motivation, and stress-coping strategies depending on the physiological state of the animal. In this context, CBD may enhance behavioral flexibility or reduce behavioral freezing under stress, resulting in increased exploratory output.

Importantly, locomotor activity in the OF test is influenced by multiple factors beyond anxiety, including novelty seeking, habituation, and general arousal. Therefore, the observed pattern may not exclusively reflect anxiety-related behavior but rather a broader alteration in the behavioral state induced by the interaction between stress and CBD. Similarly, Shu et al. (2024) did not find an influence of CBD on locomotor activity in the OF test; however, they confirmed the anxiolytic activity of CBD via force swim and tail suspension tests. Therefore, a limitation of this study is that we performed only the OF test. As shown by Shu et al. (2024), the use of multiple behavioral tests may provide more reliable results.

In contrast to the distance traveled, in the same test trial we observed significantly longer times spent in the central part of the OF test arena by animals treated with 10, 20 and 100 mg/kg CBD than by animals in the stressed nontreated group. Moreover, this parameter differed significantly between stressed and nonstressed controls (Figure 2). Time spent in the center of the OF arena is typically used as an indicator of anxiety-like behavior, with reduced center exploration and increased thigmotaxis reflecting increased anxiety (Prut and Belzung, 2003; La-Vu et al., 2020). Our results indicate anxiolytic activity of CBD at the appropriate doses since, compared with nonstressed controls, treated animals spent comparable amounts of time in the central part of the OF test arena. A low CBD dose (2 mg/kg) had no influence on time spent in the central area, suggesting that it was too low to provide an effect. In addition, high CBD doses often exhibit an inverted U-shaped dose–response curve, indicating that moderate doses may produce stronger effects than higher doses do (Zuardi et al., 2017; Uršič et al., 2022). The reason for the dose-dependent effect is that at higher doses, CBD appears to engage additional molecular targets, particularly the TRPV1 receptor, which may counteract or mask its anxiolytic actions mediated by the endocannabinoid system and serotonergic (5-HT1A) pathways (Costa et al., 2004; Wang et al., 2012; Campos et al., 2012). Consequently, very high doses can produce weaker behavioral effects than lower or intermediate doses do, a phenomenon that has been observed in both preclinical and clinical anxiety studies. This biphasic response has been reported in rodent anxiety models as well as in human studies (Zuardi et al., 2017; Narayan et al., 2022; Kwee et al., 2022; Uršič et al., 2022). However, it was not observed in our study, as the highest CBD dose (100 mg/kg) did not cause a weaker effect than lower doses (Figure 2). Although distance traveled and time spent in the center assess different aspects of OF behavior, their combined results suggest that CBD did not affect overall locomotor activity, as evidenced by comparable distance traveled between groups. However, the increased time spent in central area by CBD-treated females may indicate an anxiolytic effect of CBD.

In addition, stress response intensity was assessed by measuring plasma corticosterone concentrations. As shown in Figure 3, no significant differences were detected among any of the tested groups. In our protocol, acute stress was induced by a 1-h restraint period, which is widely used (Schmidt, 2024). Restraint, a commonly used stressor in rodents, produces an early adrenocorticotrophic hormone (ACTH) peak (typically 15–20 min after the initiation of stress). Peak corticosterone responses typically occur between 30 and 60 min and terminate within 2–3 h after onset of the stress. At 90 min after stress onset (30 min after restraint termination), corticosterone levels may have already returned to baseline levels; therefore, the absence of a robust increase at this time point does not exclude a preceding activation of the HPA axis (Herman et al., 2016). Basal plasma corticosterone concentrations can range from approximately 45 to more than 500 ng/mL, with generally higher values reported in females and concentrations influenced by the phase of estrus cycle (Hennebelle et al., 2012; Boero et al., 2022). During acute stress corticosterone concentration may increase to approximately 1,600 ng/mL or higher (Boero et al., 2022). Therefore, corticosterone concentrations measured in the present study can be considered within the basal range. Our study demonstrates that no significant differences in corticosterone concentrations were detected at the specific sampling time used (Figure 3). We believe that a single, relatively late sampling time may not adequately reflect the peak corticosterone response to acute restraint stress.

In conclusion, the present study does not support a simple anxiolytic interpretation of CBD in the OF test. Distance traveled did not differ between treated and control female rats indicating no effects of CBD treatment on locomotor activity, whereas the time spent in the central zone of the OF arena was longer in CBD-treated animals in comparison to control, suggesting anxiolytic activity of CBD. Further studies incorporating multiple behavioral assays and neurobiological markers are warranted to clarify the mechanisms underlying these observations.

## Data Availability

The original contributions presented in the study are included in the article/Supplementary Material, further inquiries can be directed to the corresponding author.
