# Supplementary material for: Protective effects of cannabidiol against acute stress: behavioral responses and corticosterone concentrations in female Wistar rats
Source: Front Pharmacol. 2026 Sep 30;17:1931847. doi: 10.3389/fphar.2026.1931847 (PMC13661023; doi:10.3389/fphar.2026.1931847)
Supplement: Supplementary file 1 [file Supplementaryfile1.pdf]

VAKOS XT a.s.
Pernerova 28a, 186 00 Praha 8, tel: +420 224 819 249, 224 814 519, fax: +420 222 327 126
e-mail: okj@vakosxt.cz; customerservice@cbdepot.eu

LL 019/0819/513	19/19 LL
číslo atestu / certificate No.	*číslo protokolu / report No.*

Analytický protokol / Certificate of Analysis

(-)-trans-Cannabidiol, GMP

(Cannabidiolum)

Číslo šarže / Batch No.:	**CBD060819**	*Počet a typ balení / Number and type of packaging:*	1 x 1 940 g
Číslo vzorku / Sample No:	LL 19/19	*Použitelné do / Best before:*	08/2021
Zkušební specifikace / Test Specification:	**PNY – LL – 01 – 17**	*Množství vzorku / Vzorkoval: Sample size / Sampled by:*	35 g / Konečný

**Název analýzy / *Type of Analysis***	**Výsledek / *Result***	**Specifikace / *Specification***
**VLASTNOSTI / *CHARACTERS***		
Vzhled / *Appearance*	**vyhovuje / *complies***	téměř bílý až nažloutlý prášek / *whitish to yellowish powder*
**ZKOUŠKY TOTOŽNOSTI / *IDENTIFICATON***		
Zkouška totožnosti *(IČ)* / *Identity (IR)*	**vyhovuje / *complies***	shoduje se s referenčním spektrem / *corresponds to the reference spektrum*
**ZKOUŠKY NA ČISTOTU / *TESTS***		
Specifická optická otáčivost / *Specific Rotation*	**-130,8**	*-129,5 to -135,0*
Příbuzné látky / *Related Substances (%)*		
Cannabinol (CBN)	< **0,05**	Cannabinol (CBN) ≤ *0,05*
Delta-9-THC	< **0,05**	Delta-9-THC ≤ *0,05*
Delta-8-THC	< **0,05**	Delta-8-THC ≤ *0,05*
Nespecifikované nečistoty jednotlivě / *Single unspecified impurities*	< **0,05**	Nespecifikované nečistoty jednotlivě / *Single unspecified impurities* ≤ *0,10*
Nečistoty celkově / *Total impurities*	< **0,5**	Nečistoty celkově / *Total impurities* ≤ *0,5*
Jiné příbuzné látky / *Other Related Substances (%)*		
Cannabidivarin (CBDV)	**0,24**	≤ *1,5*
CBD-C4	**0,2**	≤ *0,5*
Síranový popel / *Sulphated Ash (%)*	**0,05**	≤ *0,1*
Zbytková rozpouštědla / *Residual Solvents (%)* (Σ Propan-2-ol; n-Pentan, n-Heptan)	≤ **0,11**	≤ 0,5

Schválil dne / Approved on 06/09/2019

VAKOS XT a.s.
PERNEROVA 28, PRAHA 8
ODDĚLENÍ KONTROLY JAKOSTI

Ing. Josef Konečný
ředitel oddělení kontroly jakosti /
director of quality control

VAKOS XT a.s.
Pernerova 28a, 186 00 Praha 8, tel: +420 224 819 249, 224 814 519, fax: +420 222 327 126
e-mail: okj@vakosxt.cz; customerservice@cbdepot.eu

LL 019/0819/513	19/19 LL
číslo atestu / certificate No.	*číslo protokolu / report No.*

Analytický protokol / Certificate of Analysis

**Název analýzy /** ***Type of Analysis***	**Výsledek /** ***Result***	**Specifikace /** ***Specification***
**STANOVENÍ OBSAHU /** ***ASSAY***		
Cannabidiol (CBD) / *Cannabidiol (CBD) (%)*	**98,5**	*98,0 – 102,0*

Posudek / Result: **vyhovuje / *complies with* PNY – LL – 01 – 17**

The tested batch conforms to the related specification of DAC/NRF 2016/2, C-052 and of the general chapters of European Pharmacopoeia (Ph. Eur.). The mentioned batch was manufactured, analyzed, handled and stored in accordance with Good Manufacturing Practice (GMP) in conformance with the above regulations and as outlined in the International Conference on Harmonization „ICH Q7 guideline on Good Manufacturing Practice for Active Pharmaceutical Ingredients".

Schválil dne / Approved on 06/09/2019

VAKOS XT a.s.
PERNEROVA 28, PRAHA 8
ODDĚLENÍ KONTROLY JAKOSTI

....
Ing. Josef Konečný
ředitel oddělení kontroly jakosti /
director of quality control
