# Supplementary material for: Protective effects of cannabidiol against acute stress: behavioral responses and corticosterone concentrations in female Wistar rats
Source: Front Pharmacol. 2026 Sep 30;17:1931847. doi: 10.3389/fphar.2026.1931847 (PMC13661023; doi:10.3389/fphar.2026.1931847)
Supplement: Supplementary file 2 [file Supplementaryfile2.docx]

Supplementary Material 2. Homemade restraint device used to induce restraint stress. The device can be adjusted according to the size of the rat.
