# Supplementary material for: Protective effects of cannabidiol against acute stress: behavioral responses and corticosterone concentrations in female Wistar rats
Source: Front Pharmacol. 2026 Sep 30;17:1931847. doi: 10.3389/fphar.2026.1931847 (PMC13661023; doi:10.3389/fphar.2026.1931847)
Supplement: Supplementary file 3 [file Supplementaryfile3.docx]

# Supplementary material 3

**Table 1.** Results of the linear model assessing the effect of treatment group on distance travelled in the open field test.

| ***Predictors*** | ***Estimates*** | ***CI*** | ***p*** |
| --- | --- | --- | --- |
| [Intercept] | 17.62 | 13.21 – 22.04 | **<0.001** |
| Group [10 mg/kg] | 4.00 | -2.24 – 10.23 | 0.201 |
| Group [20 mg/kg] | 2.14 | -4.10 – 8.37 | 0.490 |
| Group [100 mg/kg] | 5.36 | -0.88 – 11.60 | 0.089 |
| Group [Ctrl_no] | -11.50 | -17.74 – -5.26 | **0.001** |
| Group [Ctrl_stress] | 2.12 | -4.12 – 8.36 | 0.493 |
| **Observations** | 36 | | |
| **R^2^ / R^2^ adjusted** | 0.570 / 0.498 | | |

*CI – 95 % confidence interval*

**Table 2.** Results of the generalized linear model (Gamma distribution, log link) assessing the effect of treatment group on center time in the open field test.

| ***Predictors*** | ***Estimates*** | ***CI*** | ***p*** |
| --- | --- | --- | --- |
| [Intercept] | 0.05 | 0.04 – 0.09 | **<0.001** |
| Group [10 mg/kg] | 1.81 | 0.98 – 3.36 | 0.056 |
| Group [20 mg/kg] | 2.14 | 1.16 – 3.96 | **0.014** |
| Group [100 mg/kg] | 1.35 | 0.73 – 2.50 | 0.333 |
| Group [Ctrl_no] | 1.85 | 1.00 – 3.42 | **0.049** |
| Group [Ctrl_stress] | 0.49 | 0.27 – 0.91 | **0.023** |
| **Observations** | 36 | | |
| **R^2^** **Nagelkerke** | 0.481 | | |

*CI – 95 % confidence interval*

**Table 3.** Results of the linear model assessing the effect of treatment group on plasma corticosterone concentrations.

| ***Predictors*** | ***Estimates*** | ***CI*** | ***p*** |
| --- | --- | --- | --- |
| [Intercept] | 122.67 | 93.84 – 151.49 | **< 0.001** |
| Group [10 mg/kg] | -18.83 | -59.60 – 21.93 | 0.353 |
| Group [20 mg/kg] | -13.33 | -54.10 – 27.43 | 0.509 |
| Group [100 mg/kg] | -9.50 | -50.26 – 31.26 | 0.638 |
| Group [Ctrl_no] | -41.83 | -82.60 – -1.07 | **0.045** |
| Group [Ctrl_stress] | -52.00 | -92.76 – -11.24 | **0.014** |
| **Observations** | 36 | | |
| **R^2^ / R^2^ adjusted** | 0.252 / 0.128 | | |

*CI – 95 % confidence interval*

**Table 4.** Results of the *post hoc* pairwise comparisons of distance travelled in the open field test.

| **Contrast** | **Difference** | **SE** | **DF** | **t statistic** | **Lower CI** | **Upper CI** | ***p*-value** |
| --- | --- | --- | --- | --- | --- | --- | --- |
| [2 mg/kg] - [10 mg/kg] | -4.00 | 3.05 | 30 | -1.31 | -13.29 | 5.29 | 0.778 |
| [2 mg/kg] - [20 mg/kg] | -2.14 | 3.05 | 30 | -0.70 | -11.43 | 7.15 | 0.981 |
| [2 mg/kg] - [100 mg/kg] | -5.36 | 3.05 | 30 | -1.76 | -14.65 | 3.93 | 0.508 |
| [2 mg/kg] - Ctrl_no | 11.50 | 3.05 | 30 | 3.76 | 2.21 | 20.79 | 0.009 |
| [2 mg/kg] - Ctrl_stress | -2.12 | 3.05 | 30 | -0.69 | -11.41 | 7.17 | 0.981 |
| [10 mg/kg] - [20 mg/kg] | 1.86 | 3.05 | 30 | 0.61 | -7.43 | 11.15 | 0.990 |
| [10 mg/kg] - [100 mg/kg] | -1.37 | 3.05 | 30 | -0.45 | -10.65 | 7.92 | 0.998 |
| [10 mg/kg] - Ctrl_no | 15.49 | 3.05 | 30 | 5.07 | 6.20 | 24.78 | <0.001 |
| [10 mg/kg] - Ctrl_stress | 1.88 | 3.05 | 30 | 0.61 | -7.41 | 11.17 | 0.989 |
| [20 mg/kg] - [100 mg/kg] | -3.22 | 3.05 | 30 | -1.06 | -12.51 | 6.07 | 0.895 |
| [20 mg/kg] - Ctrl_no | 13.63 | 3.05 | 30 | 4.46 | 4.34 | 22.92 | 0.001 |
| [20 mg/kg] - Ctrl_stress | 0.02 | 3.05 | 30 | 0.01 | -9.27 | 9.31 | 1.000 |
| [100 mg/kg] - Ctrl_no | 16.86 | 3.05 | 30 | 5.52 | 7.57 | 26.15 | <0.001 |
| [100 mg/kg] - Ctrl_stress | 3.24 | 3.05 | 30 | 1.06 | -6.05 | 12.53 | 0.893 |
| Ctrl_no - Ctrl_stress | -13.62 | 3.05 | 30 | -4.46 | -22.91 | -4.33 | 0.001 |

*SE – standard error*

*DF – degrees of freedom*

*CI – 95 % confidence interval*

**Table 5.** Results of the *post hoc* pairwise comparisons of center time spent in the open field test.

| **Contrast** | **Ratio** | **SE** | **DF** | **t statistic** | **Lower CI** | **Upper CI** | ***p-*value** |
| --- | --- | --- | --- | --- | --- | --- | --- |
| [2 mg/kg] / [10 mg/kg] | 0.560 | 0.169 | 30 | -1.92 | 0.22 | 1.40 | 0.4111 |
| [2 mg/kg] / [20 mg/kg] | 0.476 | 0.144 | 30 | -2.46 | 0.19 | 1.19 | 0.1685 |
| [2 mg/kg] / [100 mg/kg] | 0.747 | 0.225 | 30 | -0.97 | 0.30 | 1.87 | 0.9245 |
| [2 mg/kg] / Ctrl_no | 0.550 | 0.166 | 30 | -1.98 | 0.22 | 1.38 | 0.3777 |
| [2 mg/kg] / Ctrl_stress | 1.957 | 0.591 | 30 | 2.22 | 0.78 | 4.90 | 0.2570 |
| [10 mg/kg] / [20 mg/kg] | 0.849 | 0.256 | 30 | -0.54 | 0.34 | 2.13 | 0.9939 |
| [10 mg/kg] / [100 mg/kg] | 1.332 | 0.402 | 30 | 0.95 | 0.53 | 3.34 | 0.9296 |
| [10 mg/kg] / Ctrl_no | 0.982 | 0.297 | 30 | -0.06 | 0.39 | 2.46 | 1.0000 |
| [10 mg/kg] / Ctrl_stress | 3.492 | 1.054 | 30 | 4.14 | 1.39 | 8.75 | 0.0032 |
| [20 mg/kg] / [100 mg/kg] | 1.569 | 0.474 | 30 | 1.49 | 0.63 | 3.93 | 0.6717 |
| [20 mg/kg] / Ctrl_no | 1.157 | 0.349 | 30 | 0.48 | 0.46 | 2.90 | 0.9965 |
| [20 mg/kg] / Ctrl_stress | 4.112 | 1.242 | 30 | 4.68 | 1.64 | 10.30 | 0.0007 |
| [100 mg/kg] / Ctrl_no | 0.737 | 0.223 | 30 | -1.01 | 0.29 | 1.85 | 0.9108 |
| [100 mg/kg] / Ctrl_stress | 2.621 | 0.791 | 30 | 3.19 | 1.05 | 6.57 | 0.0354 |
| Ctrl_no / Ctrl_stress | 3.556 | 1.073 | 30 | 4.20 | 1.42 | 8.91 | 0.0027 |

*SE – standard error*

*DF – degrees of freedom*

*CI – 95 % confidence interval*

**Table 6.** Results of the *post hoc* pairwise comparisons of plasma corticosterone concentrations.

| **Contrast** | **Difference** | **SE** | **DF** | **t statistic** | **Lower CI** | **Upper CI** | ***p*-value** |
| --- | --- | --- | --- | --- | --- | --- | --- |
| [2 mg/kg] - [10 mg/kg] | 18.83 | 19.96 | 30 | 0.94 | -41.88 | 79.54 | 0.932 |
| [2 mg/kg] - [20 mg/kg] | 13.33 | 19.96 | 30 | 0.67 | -47.38 | 74.04 | 0.984 |
| [2 mg/kg] - [100 mg/kg] | 9.50 | 19.96 | 30 | 0.48 | -51.21 | 70.21 | 0.997 |
| [2 mg/kg] - Ctrl_no | 41.83 | 19.96 | 30 | 2.10 | -18.88 | 102.54 | 0.316 |
| [2 mg/kg] - Ctrl_stress | 52.00 | 19.96 | 30 | 2.61 | -8.71 | 112.71 | 0.127 |
| [10 mg/kg] - [20 mg/kg] | -5.50 | 19.96 | 30 | -0.28 | -66.21 | 55.21 | 1.000 |
| [10 mg/kg] - [100 mg/kg] | -9.33 | 19.96 | 30 | -0.47 | -70.04 | 51.38 | 0.997 |
| [10 mg/kg] - Ctrl_no | 23.00 | 19.96 | 30 | 1.15 | -37.71 | 83.71 | 0.855 |
| [10 mg/kg] - Ctrl_stress | 33.17 | 19.96 | 30 | 1.66 | -27.54 | 93.88 | 0.566 |
| [20 mg/kg] - [100 mg/kg] | -3.83 | 19.96 | 30 | -0.19 | -64.54 | 56.88 | 1.000 |
| [20 mg/kg] - Ctrl_no | 28.50 | 19.96 | 30 | 1.43 | -32.21 | 89.21 | 0.710 |
| [20 mg/kg] - Ctrl_stress | 38.67 | 19.96 | 30 | 1.94 | -22.04 | 99.38 | 0.400 |
| [100 mg/kg] - Ctrl_no | 32.33 | 19.96 | 30 | 1.62 | -28.38 | 93.04 | 0.592 |
| [100 mg/kg] - Ctrl_stress | 42.50 | 19.96 | 30 | 2.13 | -18.21 | 103.21 | 0.300 |
| Ctrl_no - Ctrl_stress | 10.17 | 19.96 | 30 | 0.51 | -50.54 | 70.88 | 0.995 |

*SE – standard error*

*DF – degrees of freedom*

*CI – 95 % confidence interval*
